# Effects of Cortisol Administered through Slow-Release Implants on Innate Immune Responses in Rainbow Trout (*Oncorhynchus mykiss*)

**DOI:** 10.1155/2013/619714

**Published:** 2013-08-29

**Authors:** R. Cortés, M. Teles, R. Trídico, L. Acerete, L. Tort

**Affiliations:** ^1^Department of Cell Biology, Physiology and Immunology, Autonomous University of Barcelona, 08193 Barcelona, Spain; ^2^Department of Fish Physiology and Biotechnology, Institute of Aquaculture Torre de la Sal, Spanish National Research Council (IATS-CSIC), Ribera de Cabanes, 12595 Castellón, Spain; ^3^Institute of Biotechnology and Biomedicine, Autonomous University of Barcelona, 08193 Barcelona, Spain

## Abstract

Cortisol is a key hormone in the fish stress response with a well-known ability to regulate several physiological functions, including energy metabolism and the immune system. However, data concerning cortisol effects on fish innate immune system using a more controlled increase in cortisol levels isolated from any other stress related signaling is scarce. The present study describes the effect of doses of cortisol corresponding to acute and chronic levels on the complement and lysozyme activity in plasma of the rainbow trout (*Oncorhynchus mykiss*). We also evaluated the effects of these cortisol levels (from intraperitoneally implanted hydrocortisone) on the mRNA levels quantified by RT-qPCR of selected key immune-related genes in the liver, head kidney, and spleen. For that purpose, 60 specimens of rainbow trout were divided in to two groups: a control group injected with a coconut oil implant and another group injected with the same implant and cortisol (50 **μ**g cortisol/g body weight). Our results demonstrate the role of cortisol as a modulator of the innate immune response without the direct contribution of other stress axes. Our results also show a relationship between the complement and lysozyme activity in plasma and mRNA levels in liver, supporting the important role of this organ in producing these immune system proteins after a rise of cortisol in the fish plasma.

## 1. Introduction

In fish, as in mammals, the innate immune system is the first line of defense against infection that acts by recognizing and attacking nonspecifically the pathogens and helping to maintain homeostasis and health [1–3]. Among its components, some plasma proteins, such as complement components and lysozyme, are of primary importance. These humoral proteins act by lysing bacteria [4, 5] and it has been demonstrated that they are in the core response of fish immunity [6] and that these responses are mediated by cytokines [7, 8].

The response of the innate immune system may be modified by external stimuli, particularly stressors, which most often are known to induce immunosuppressive effects in fish [7, 9, 10]. Thus, it has been shown that stressed fish have decreased activity of complement, hemagglutination, and reduction of circulating lymphocytes [11, 12]. Decreases in C3 complement component protein levels [13] and decrease in expression of immune-relevant genes [14, 15] have also been found.

Generally, the immunosuppressive consequences of stressors are attributed to the action of circulating glucocorticoids, in particular cortisol. Both in mammals and fish, cortisol exerts powerful anti-inflammatory effects, inhibiting inflammatory mediators including cytokines [10, 16]. *In vitro* studies using primary cell cultures of head kidney showed that cortisol effects on immune parameters are mainly suppressive [17, 18]. Moreover, microarray analysis after *in vivo* treatments with cortisol implants showed that this hormone modulates the expression of immune-related genes responsible for antigen recognition, antiviral activity, and inflammatory responses [19, 20].

Lysozyme takes also part in an extensive battery of defense mechanisms in fish, such as bacteriolysis and opsonization of the bacterial wall, and it is present in lymphoid tissues, mucus, plasma, and other body fluids [2]. The complement system is composed of at least 35 soluble proteins that are responsible for the activation of the membrane attack complex and lytic activity. Amongst them, the C3 protein directly binds to the surface of the antigen [21], the B factor (Bf) is attached to the bacterial wall, and both C3b and Bf form the complex C3-convertase, whose role is splitting in more C3 molecules, thereby producing a sequential amplification [22]. The H factor of the complement (Hf) has the function to attach to C3b-Bf complex displacing Bf protein, stopping the response activated by C3b-convertase complex [23].

Cytokines regulate the inflammatory response which is responsible for protecting the body against invading pathogens and also preventing the cellular damage to maintain homeostasis. Proinflammatory cytokines such as tumor necrosis factor-*α* (TNF-*α*), interleukin-1*β* (IL-1*β*), and interleukin-6 (IL-6) initiate defensive mechanisms in response to microorganisms and antigens present in the body [24]. Their activity must be well controlled, as any inactivation or an excessive response can lead to dysfunction in antigen control. In order to avoid this, cytokines such as transforming growth factor-*β* (TGF-*β*) are responsible for terminating and preventing an exaggerated response, inhibiting, for example, the action of TNF-*α* [18, 25]. Previous findings on the effects of cortisol on *in vitro* cell lines in gilthead seabream and rainbow trout found that stimulation of macrophages with cortisol caused a decrease in the expression of proinflammatory cytokines [17, 18]. Similar results showing reduction of cytokine expression have been reported after *in vivo* treatments in atlantic salmon [19] and gilthead sea bream [20].

Considering the previous findings, we aimed to evaluate to what extent cortisol acts as an immunomodulator on selected nonspecific immune responses such as complement and lysozyme and on the mRNA levels of some key related genes in rainbow trout. We analyzed the effects in the liver, head kidney, and spleen by using cortisol implants, thus focusing on the effects of cortisol itself [26, 27] and complementing the existing data obtained after *in vitro* or husbandry stress studies. Cortisol implants have been previously shown to produce a slow release of cortisol into the circulation [28]. Thus, we decided to use cortisol implants since our aim was to create medium to high levels of circulating of this hormone over extended time periods emulating cortisol release after acute and chronic stress.

## 2. Materials and Methods

### 2.1. Animals

*Oncorhynchus mykiss* (167 ± 10 g mean weight) were obtained from a local fish farm (Piscifactoria Andrés, St. Privat d'en Bas, Spain). Fish were acclimatized to laboratory conditions for 15 days, maintained in a closed freshwater recirculation system, at 15°C, in a 12 h light/12 h dark cycle and fed a commercial diet once a day. The fish density in the aquarium was 8.4 kg/m^3^. Fish were fasted 24 h before the experimental procedure. Dissolved oxygen, pH, nitrite, nitrate, and ammonia were analyzed periodically. All procedures were conducted according to ethical guidelines of animal experimentations (CEE 86/609 regulation) and they were supervised by the ethical committee of the Universitat Autònoma de Barcelona.

### 2.2. Experimental Design and Sampling Procedure

Fish were slightly anaesthetized with 0.1 g/L of tricaine methane sulphonate (MS222), intraperitoneally (ip) injected with implants of either coconut oil alone (control) or coconut oil containing 50 *μ*g cortisol/g body weight (bw) (hydrocortisone hemisuccinate), and immediately returned to their respective tanks. After 1, 5, and 10 days of cortisol implantation fish were randomly captured and sacrificed by overanesthetization with MS222 (1 g/L).

Blood was collected with heparinized syringes from the caudal vein and used for plasma isolation using an Eppendorf centrifuge. The overall blood collection lasted less than 3 minutes in order to avoid cortisol rise induced by the manipulation during sampling. Liver, head kidney, and spleen were carefully dissected out and immediately frozen in liquid nitrogen for the transcriptional analysis. All samples were stored at −80°C until analysis.

### 2.3. Plasma Complement and Lysozyme Analysis

Activity of the alternative complement pathway (ACP) was determined following the technique described by Sunyer et al. [29] with minor modifications for ELISA plates [30]. Lysozyme activity assays (kU/mL) were performed by a turbidimetric method that uses the lysis of *Micrococcus luteus* (Sigma) for determination of the lysozyme activity using egg-white lysozyme as standard.

### 2.4. Gene Expression Quantification

Total RNA was individually extracted from tissues using 1 mL per sample of TRI reagent following the manufacturer's instructions. RNA quantification was carried out using a NanoDrop ND-1000 (Thermo Scientific) and the quality of the RNA checked with the Experion RNA StdSens Analysis Kit (Bio-Rad). All RIN (RNA integrity number) values obtained were above 8, indicative of excellent RNA integrity and quality. RNA (4 *μ*g) was used to synthesize complementary DNA (cDNA) with Superscript III reverse transcriptase (Invitrogen) and Oligo-dT primer (Promega). cDNA was used as a template for quantitative PCR transcript quantification. Amplifications were carried out in 20 *μ*L reaction volume, containing 10 *μ*L of SYBR Green Supermix (Bio-Rad), 5 *μ*L of cDNA in a dilution of 1 : 50 for the target genes, and 500 nM of each primer. 18S rRNA was used as a housekeeping gene in a dilution of 1 : 10000. All samples were run in triplicate in the iCycler iQ Thermal Cycler (Bio-Rad) under the following conditions: initial denaturation at 95° for 5 min followed by 40 cycles of 95°C/10 s, 60°C/30 s, 1 cycle of 95°C for 1 min, and 1 cycle of 55°C for 1 min. The melting curve conditions were as follows: starting temperature 55°C and it was increased 0.5°C in each step; the dwelling time was 30′′. At the end of the step the fluorescence was measured. The final temperature was 95°C. When analyzing the melting curve we observed that no contaminating products were present and no primer dimers were formed during the reactions. Reactions lacking cDNA and containing RNA were used as negative controls. Values were expressed as fold changes; Ct were normalized to 18S expression and analyzed according to the Pfaffl method corrected for efficiency for each primer set relative to controls [31]. For this study, we choose 18S rRNA as a reference gene since it was previously validated in our laboratory, as well as in the present experiment. Primer sets for real-time quantitative PCR (qPCR) (Table 1) were designed with Primer3 version 4.0 based on target sequences obtained from the rainbow trout database and analyzed with IDT OligoAnalyzer 3.1.

### 2.5. Data Analysis

IBM SPSS Statistics 20 software was used for statistical analyses. For plasma parameters, differences between control and implanted groups were tested using the two-tailed Student's *t*-test and the significance level was fixed at *P* < 0.05. For gene expression the differences were tested using one-way ANOVA, followed by HSD *post hoc* test (*P* < 0.05) comparing the results of mRNA levels obtained in each one of the experimental groups at days 1, 5, and 10 with the control group. The experiments were carried out using test groups of six fish (*n* = 6).

## 3. Results and Discussion

The cortisol implanted groups had statistically significant elevated plasma cortisol levels after 1 and 5 days (274 ng/mL and 80 ng/mL, resp.) after implantation when compared to their respective controls. After 10 days of implantation plasma cortisol was 27.7 ng/mL, meaning that plasma cortisol returned to basal levels, as this value was not significantly different from controls [20, 32]. The increase in cortisol levels after implantation were within a physiological and stress-relevant range allowing to study both acute and chronic effect of elevated cortisol levels.

**Figure 1:**
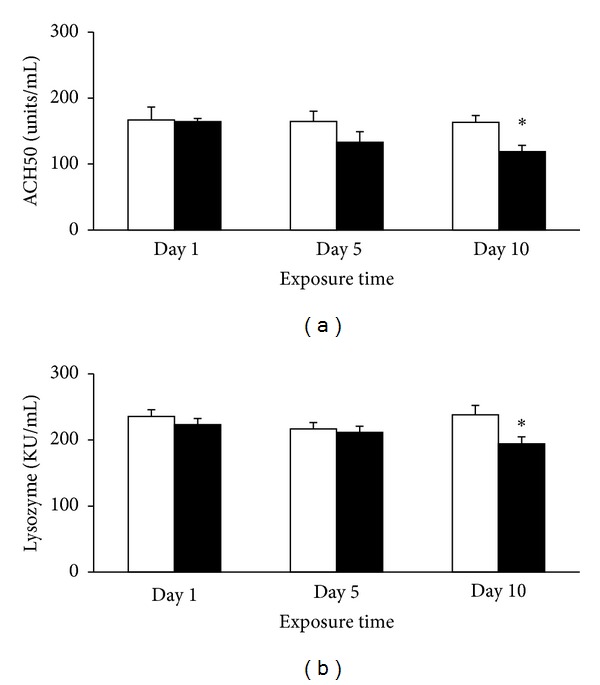
Plasma complement ACH50 (a) and lysozyme (b) activities of *O. mykiss* 1, 5, and 10 days after ip implantation of 5 *μ*L/g body weight (bw) of coconut oil alone (control, open bars) and containing 50 *μ*g/g bw cortisol (closed bars). Values represent the means and SEM (*n* = 6 per group). Significant differences are highlighted as ∗*versus* control (*P* < 0.05).

Complement activity results were expressed as the titre at which 50% hemolysis is produced (ACH50). At day 1 after cortisol implantation, ACH50 (Figure 1(a)) and lysozyme (Figure 1(b)) activities showed no differences compared to control. At day 5 after implantation, when the cortisol levels were still increased, plasma ACH50 and lysozyme slightly decreased, though not significantly. When plasma cortisol levels returned to basal levels at day 10, we found significantly decreased activities of ACH50 and lysozyme in the plasma. These results reflect a latter downregulatory effect of cortisol on the activity of both ACH50 and lysozyme. These results are in agreement with previous findings in stressed fish, where both repeated acute and chronic stressors induced a decrease in plasma activities of complement and lysozyme [29, 33, 34].

**Figure 2:**
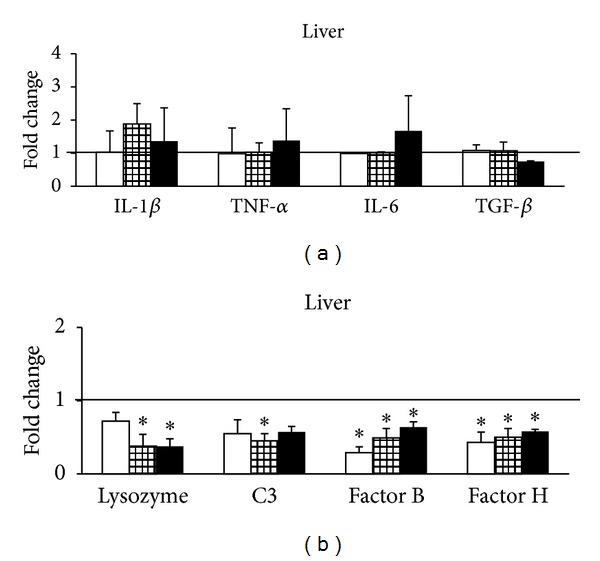
Real-time RT-qPCR analysis of IL-1*β*, TNF-*α*, IL-6, TGF-*β* (a), lysozyme, C3, factor B, and factor H (b) in the liver of *O. mykiss* 1 (open bars), 5 (gridded bars), and 10 (closed bars) days after ip implantation of coconut oil alone (control) or containing 50 *μ*g/g bw cortisol. Relative expression levels were normalized to 18S rRNA and fold changes were calculated against control. Values represent the means and SEM (*n* = 6 per group). Significant differences are highlighted as ∗*versus* control (*P* < 0.05). The horizontal line originating at *y* = 1 denotes the control group against which the expression was compared.

Analysis of mRNA levels in the liver (Figure 2(a)) revealed that cytokine expression showed no differences compared to control levels. This result was according to expectations since short term changes in cytokines can be expected mainly in immune-related organs [35–37], whereas changes related to energetic and intermediary metabolism would show variations at medium and longer terms [27, 33]. Lysozyme mRNA abundance was unaltered at day 1 after cortisol implantation. However, it was significantly decreased afterwards. The C3 mRNA abundance was significantly decreased at day 5 after cortisol implantation, while at days 1 and 10 it showed control levels. Bf and Hf mRNA levels were significantly decreased in the liver throughout experiment (Figure 2(b)). These changes in specific mRNA abundance correlate well with the plasma data, since both lysozyme and complement activities decreased. In rainbow trout the liver is the main source of these humoral components, as previously reported by Løvoll et al. [36] for C3 and Bf. Thus, present results suggest that cortisol regulates the expression of lysozyme and complement factors in the liver and that this effect would possibly lead to reduced plasma ACH50 and lysozyme activities. A similar situation occurred with other genes of the immune system in the liver of gilthead sea bream (*Sparus aurata*) implanted intraperitoneally with cortisol [20].

**Figure 3:**
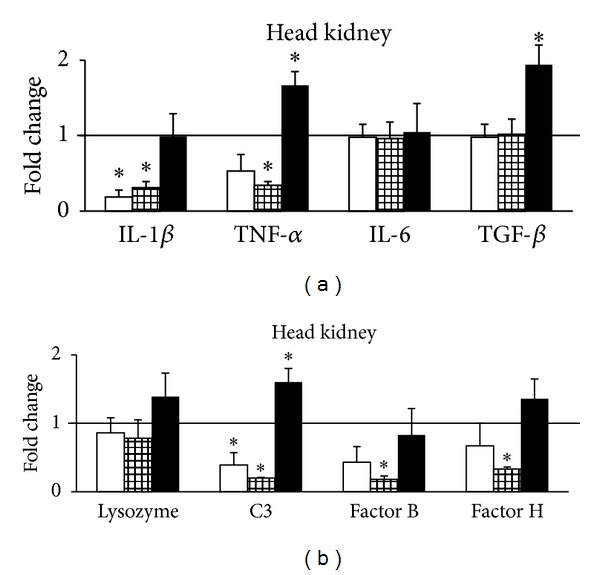
Real-time RT-qPCR analysis of IL-1*β*, TNF-*α*, IL-6, TGF-*β* (a), lysozyme, C3, factor B, and factor H (b) in the head kidney of *O. mykiss* 1 (open bars), 5 (gridded bars), and 10 (closed bars) days after ip implantation of coconut oil alone (control) or containing 50 *μ*g/g bw cortisol. Relative expression levels were normalized to 18S rRNA and fold changes were calculated against control. Values represent the means and SEM (*n* = 6 per group). Significant differences are highlighted as ∗*versus* control (*P* < 0.05). The horizontal line originating at *y* = 1 denotes the control group against which the expression was compared.

In the head kidney we found a significant downregulation of IL-1*β* mRNA levels at days 1 and 5 after cortisol implantation (Figure 3(a)). TNF-*α* expression levels showed a decreasing trend though not significant at day 1 after implantation becoming significant at day 5. These results show the modulatory action of cortisol on the expression of these proinflammatory cytokines. Moreover, it is likely that a downregulation of TNF-*α* due to high plasma cortisol levels induces a decrease in IL-1*β* mRNA levels, thus showing immune-suppressive effects of cortisol over the transcriptional levels of proinflammatory genes. Hence, it has been shown in mammals that the secretion of TNF-*α* stimulates the production of IL-1*β* during the inflammatory response [38, 39]. At day 10, when plasma cortisol levels were again basal, IL-1 *β* expression levels were unaltered and TNF-*α* expression levels were significantly increased. Nevertheless, an interplay of cortisol from plasma coming from the implant release with cortisol from paracrine or autocrine sources from local internal cells cannot be discarded.

IL-6 expression levels did not change during the 10 days of the experiment (Figure 3(a)) indicating that under the present experimental circumstances this cytokine was not affected by cortisol action. This finding may indicate that IL-6 expression is probably not mediated by hormonal action, as suggested by Chettri et al. [40]. However, this contrasts with previous results in cell cultures of head kidney, in which IL-6 downregulation was found [17, 18]. TGF-*β* mRNA abundance was unaltered both at days 1 and 5 after cortisol implantation (Figure 3(a)). Castro et al. [18] also observed unaltered TGF-*β* mRNA levels in rainbow trout cell cultures stimulated with cortisol. However, at day 10 the expression levels of TGF-*β* significantly increased, probably as a way of controlling the previous upregulation of TNF-*α* [35]. Lysozyme mRNA abundance was unaltered in the head kidney (Figure 3(b)). The complement component C3 was significantly downregulated both at day 1 and at day 5 after cortisol implantation. However, at day 10, C3 mRNA abundance was significantly upregulated. Bf and Hf mRNA abundance was significantly downregulated at day 5 after cortisol implantation but unaltered at days 1 and 10. In the head kidney, plasma cortisol modulated negatively the complement factors at days 1 and 5. At day 10, when plasma cortisol levels were basal and plasma complement activity decreased, Bf and Hf presented unaltered expression levels, and C3 mRNA abundance increased. These results show that there is a delayed response in terms of plasma complement activity compared to the transcriptional pattern observed in the organ. However, one cannot disregard that the measured plasma ACH50 is the sum of the action of the complement protein cascade, and not only of C3. Therefore, it is likely that during stressful events, the elevated circulating cortisol levels can modulate the expression of genes related to the complement pathway, such as C3. Such an effect can explain a higher susceptibility to pathogens in stressed fish, as the activity of the complement system would be suppressed [41]. 

**Figure 4:**
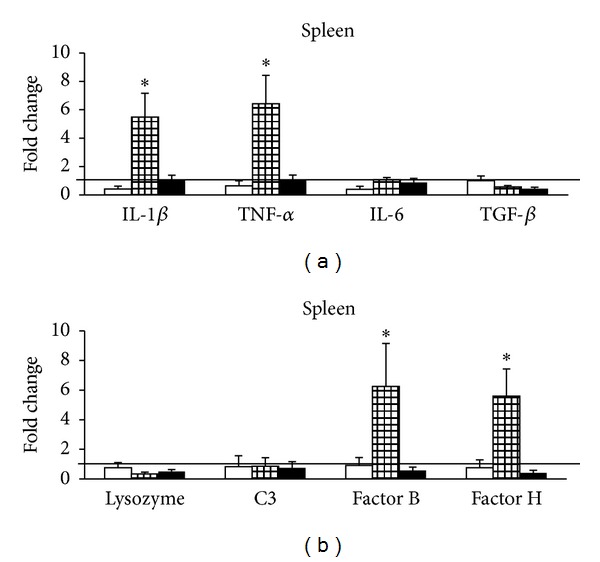
Real-time RT-qPCR analysis of IL-1*β*, TNF-*α*, IL-6, TGF-*β* (a), lysozyme, C3, factor B, and factor H (b) in the spleen of *O. mykiss* 1 (open bars), 5 (gridded bars), and 10 (closed bars) days after ip implantation of coconut oil alone (control) or containing 50 *μ*g/g bw cortisol. Relative expression levels were normalized to 18S rRNA and fold changes were calculated against control. Values represent the means and SEM (*n* = 6 per group). Significant differences are highlighted as ∗*versus* control (*P* < 0.05). The horizontal line originating at *y* = 1 denotes the control group against which the expression was compared.

In the spleen, IL-1*β* and TNF-*α* mRNA abundance significantly increased at day 5 after cortisol implantation and remained unaltered both at day 1 and at day 10 of the experiment. IL-6, TGF-beta (Figure 4(a)), lysozyme, and complement C3 mRNA abundance (Figure 4(b)) were unaltered throughout the experiment in the spleen. However, Bf and Hf mRNA abundance was significantly increased at day 5 after cortisol implantation when compared to control, but unaltered at days 1 and 10 (Figure 4(b)). These results could indicate that just as it occurs in higher vertebrates, spleen plays a filtering role inducing the increased expression levels of IL-1*β*, TNF-*α*, and complement components on day 5 after implantation due to the accumulation of the leukocytes during this filtering activity [37].

Taking together the results in these three tissues it may be suggested that on one hand the response of each tissue is different but on the other hand the time course of the cortisol effects would be specific for each one. Thus, cortisol injected to the peritoneum would reach the head kidney and exert its effects (mostly suppressive) there. After 5 days we see significant effects in spleen, probably related to leukocyte migration derived from the initial cortisol action at the head kidney where cortisol is synthesized and released and where hematopoiesis is also produced. Thus, these results also support previous data regarding the time-course effect of cortisol on corticosteroid receptors expression in fish tissues [32].

Overall, the present results support the relevant role of cortisol in modulating key components of the innate immune response in fish as shown by both the circulating protein levels of plasma lysozyme and complement components and the mRNA expression of C3, Bf, Hf, and lysozyme.

## Figures and Tables

**Table 1:** Sequences of primers used in gene expression analysis and accession numbers.

Gene	Forward primer	Reverse primer	A. number
IL-1*β*	CGTCACTGACTCTGAGAACAAGT	TGGCGTGCAGCTCCATAG	AJ223954
IL-6	TTTCAGAAGCCCGTGGAAGAGA	TCTTTGACCAGCCCTATCAGCA	DQ866150
TNF-*α*	CGCTGACACAGTGCAGTGGA	TCCCCGATGGAGTCCGAATA	NM_001124357.1
TGF-*β*	ACGGCTCAACCTGAATATGG	CGCACACAGCAACTCTCC	X99303.1
Lysozyme	TGCCTGTCAAAATGGGAGTC	CAGCGGATACCACAGACGTT	X59491
C3	GAGATGGCCTCCAAGAAGATAGAA	ACCGCATGTACGCATCATCA	L24433.1
Factor H	TGGTCACCTTCCACAACAGATT	TGACCCCGCTGCCAGTAG	NM_001124410
Factor B	CACCAAAAAGAAGACCTCCTCAA	TACAGGCAAGACAGAACCACTTTC	NM_001124595
18S	CGAGCAATAACAGGTCTGTG	GGGCAGGGACTTAATCAA	AF243428.2
